# Competitiveness-enhancing pathogen virulence gene expression and associated inducing molecules in human urine

**DOI:** 10.1186/1753-6561-9-S1-A21

**Published:** 2015-01-14

**Authors:** NGD O’Mara, MB Prentice

**Affiliations:** 1 School of Medicine University College Cork, Western Road, Cork, Ireland; 2 Departments of Pathology/Microbiology, University College Cork, Western Road, Cork, Ireland

## Background

The abundance of ethanolamine (EA) and 1,2-propanediol (PD) within the mammalian intestine has recently been hypothesized to provide certain pathogenic bacteria with a niche-specific carbon/nitrogen and energy source and provide a signal to enteric pathogens of their arrival in the small intestine. PD and EA metabolism may enhance competitive advantage for pathogen growth in other body compartments where these compounds are present. Pathogens such as Salmonella, Escherichia coli and Klebsiella utilise ethanolamine, while propanediol usage occurs in Yersinia, Klebsiella, Salmonella and Clostridium [2][3]. These pathogens possess the pdu and/or eut operon(s), which encode the necessary metabolic machinery to utilise PD/EA in addition to a number of virulence genes that may be induced by pdu/eut regulatory genes. In a preliminary study, we detected PD and EA in human urine, demonstrated that urinary pathogens can metabolise these molecules in vitro and observed growth of bacteria possessing pdu/eut operons in human urine.

## Methods

Over a 10-month period 70 urine samples were obtained from the Bacteriology laboratory at Cork University Hospital, half of which were from patients with coliform-type urinary tract infections. Gas chromatography and liquid chromatography mass spectrometry methods were used to quantify PD and EA respectively in a cohort of 20 urine samples. Chromogenic media (PD-enriched MacConkey agar) was utilised to demonstrate bacterial PD metabolism in bacteriuric samples. Using a Escherichia coli ECOR library and K. pneumoniae strain (NCIMB 132128), 18-hour kinetic growth studies of known pdu/eut positive bacteria in human urine were performed.

## Results

Growth studies revealed that eut/pdu positive bacteria grew well within human urine samples whether or not urine was supplemented with PD and EA. PD was determined to be present in all tested urine samples (n=19, 10 infected, 9 non-infected) in varying concentrations (trace to 8.8mM), while EA was present in much smaller quantities (trace to 0.13mM). PD metabolism was demonstrated in two putative Klebsiella spp. bacteriuric isolates (n=15).

## Conclusions

EA and PD are detectable and present within human urine PD is present in larger amounts. PD utilisation is known to occur in a minority of urinary pathogens. Quantitative gene expression studies will be used to seek pdu/eut operon expression from urinary isolates

## References

[B1] Garsin D Ethanolamine utilization in bacterial pathogens: roles and regulation Nature Reviews Microbiology 2010 8 4 290 295 20234377 10.1038/nrmicro2334 PMC2950637

[B2] Bobik TA The propanediol utilization (pdu) operon of Salmonella enterica serovar Typhimurium LT2 includes genes necessary for formation of polyhedral organelles involved in coenzyme B12- dependent 1,2 -propanediol degradation Journal of Bacteriology 1999 181 19 5967 5975 10498708 10.1128/jb.181.19.5967-5975.1999 PMC103623

[B3] Bertin Y EHEC gains a competitive advantage by using ethanolamine as a nitrogen source in the bovine intestinal content Environmental Microbiology 2011 13 2 365 77 20849446 10.1111/j.1462-2920.2010.02334.x

